# Development of an Ultra-Low Carbon MgO Refractory Doped with α-Al_2_O_3_ Nanoparticles for the Steelmaking Industry: A Microstructural and Thermo-Mechanical Study

**DOI:** 10.3390/ma13030715

**Published:** 2020-02-05

**Authors:** C. Gómez-Rodríguez, G. A. Castillo-Rodríguez, E. A. Rodríguez-Castellanos, F. J. Vázquez-Rodríguez, J. F. López-Perales, J. A. Aguilar-Martínez, D. Fernández-González, L. V. García-Quiñonez, T. K. Das-Roy, L. F. Verdeja

**Affiliations:** 1 Facultad de Ingeniería Mecánica y Eléctrica (FIME), Universidad Autónoma de Nuevo León (UANL), San Nicolás de los Garza 66450 N.L., Mexico; alan.castillo.rdz@gmail.com (G.A.C.-R.); earc22@hotmail.com (E.A.R.-C.); fcofimeuanl@gmail.com (F.J.V.-R.); lopez_perales_jesus@hotmail.com (J.F.L.-P.); josue.aguilar74@gmail.com (J.A.A.-M.); fetudas@prodigy.net.mx (T.K.D.-R.); 2 Department of Materials Science and Metallurgical Engineering, School of Mines, Energy and Materials, University of Oviedo, 33003 Oviedo/Uviéu, Spain; lfv@uniovi.es; 3 CONACYT-Centro de Investigación Científica y de Educación Superior de Ensenada (CICESE), Monterrey km, 9.5 Nueva Carretera al Aeropuerto, PITT Apodaca, Nuevo León 66603, Mexico; adnilanavivi_1984@hotmail.com

**Keywords:** magnesia, refractories, α-Al_2_O_3_ nanoparticles, magnesium-alumina spinel, sintering

## Abstract

The effect of α-Al_2_O_3_ nanoparticles (up to 5 wt.%) on the physical, mechanical, and thermal properties, as well as on the microstructural evolution of a dense magnesia refractory is studied. Sintering temperatures at 1300, 1500, and 1600 °C are used. The physical properties of interest were bulk density and apparent porosity, which were evaluated by the Archimedes method. Thermal properties were examined by differential scanning calorimetry. The mechanical behavior was studied by cold crushing strength and microhardness tests. Finally, the microstructure and mineralogical qualitative characteristics were studied by scanning electron microscopy and X-ray diffraction, respectively. Increasing the sintering temperature resulted in improved density and reduced apparent porosity. However, as the α-Al_2_O_3_ nanoparticle content increased, the density and microhardness decreased. Microstructural observations showed that the presence of α-Al_2_O_3_ nanoparticles in the magnesia matrix induced the magnesium-aluminate spinel formation (MgAl_2_O_4_), which improved the mechanical resistance most significantly at 1500 °C.

## 1. Introduction

Since the introduction of magnesia (MgO), its use as a basic refractory has tremendously increased due to its reasonable cost, excellent chemical resistance to basic slags and fluxes at high temperatures, as well as a high melting point (2800 °C). These properties have made MgO-based refractories preferred by the iron, non-ferrous, and cement industries [1,2,3,4]. The iron industries have widely used magnesia in the steel-making process, where it is mainly applied in steel converters, electric arc furnaces, and ladle linings. However, the thermal conductivity and high thermal expansion coefficient of MgO are affected in such a way by high temperatures to induce thermal spalling under heating conditions [5]. This effect has been mitigated since the development of magnesia-carbon (MgO-C) refractory in the 1970s, whereby the thermal properties of magnesia have been controlled and improved. A high thermal conductivity, excellent thermal shock resistance, as well as good corrosion resistance can be achieved in this kind of refractory [6,7,8,9,10,11,12,13]. Therefore, the mechanical and chemical properties exhibited by carbon-containing refractories have allowed them to be widely used to form specific compounds for certain applications in the steel industry.

Initially, MgO-C refractories were manufactured from high purity MgO clinker together with graphite flakes (carbon contents 86–99 wt.%), black carbon, and coke binders. The carbon content in conventional carbon-containing refractories ranges between 5 and 20 wt.% [10,14,15].

Nevertheless, serious drawbacks can ensue with higher carbon content, such as (i) oxidation of carbon results in a highly porous structure with weak bonding, poor mechanical strength that allows easy penetration, and eventual corrosion by slag and molten steel; (ii) increased shell temperature; (iii) higher energy consumption originated by increased conductivity of the refractory; (iv) release of carbon dioxide or carbon monoxide gases; and (v) difficulty in precisely controlling the carbon content in steel [5,13,16,17,18,19]. Certainly, the particular sensitivity of carbon with respect to oxygen is the major defect of MgO-C refractories.

To overcome the oxidation phenomenon, researchers have been investigating the use of different oxides and non-oxide antioxidants such as Al, Si, Mg, Al_8_B_4_C_7_, SiC, SiB_4_, CaB_6_, ZrO_2_, CaO, MgAl_2_O_4_, and Al_2_O_3_ in order to increase the oxidation resistance of MgO-C refractories [20,21,22,23,24,25,26,27,28,29,30,31,32,33,34,35,36,37].

For some years now, the steel industry has demanded the clean production of steel in terms of energy savings, emission reductions, and pollution of molten steel by spalling carbon from the refractory (altering the chemical composition of the steel). Given this and considering that conventional carbon-containing refractories do not meet the necessary requirements, researchers have been encouraged to design and implement refractories with low and ultra-low carbon content. However, as the carbon content is reduced in the MgO-C refractories, mechanical and thermal properties (e.g., thermal shock resistance) are affected [14,38,39,40].

Faced with the challenge of obtaining refractories with low and ultra-low carbon contents but exhibiting excellent thermo-mechanical properties, graphite flakes have been replaced in recent years by micro and nano-sized carbon particles.

Nanotechnology is currently used in many research applications with outstanding results [41,42,43,44,45,46,47,48,49,50,51,52,53,54,55,56,57,58,59,60,61,62,63,64,65]. Therefore, nanocarbon sources such as [47,48,49,50,51,52,53] black (CB), nanofibers (CNFs), nanotubes (CNTs), expanded graphite (EG), and graphene or graphite oxide nanosheets (GONs) have been used in the development of MgO-C refractories with low and ultra-low carbon content.

Wei et al. [54] studied the effect of adding Fe nanosheets (from 0 to 1.0 wt.%) to the microstructure of low-carbon MgO–C refractories bonded with phenol resin. They found that the mechanical and thermal shock resistances of low-carbon refractories with 0.5 wt.% Fe nanosheets are highly improved in comparison with specimens without Fe nanosheets, which is attributed to the in situ formation of CNTs and the appearance of bridges that induce a crack deflection mechanism in the matrix. Matsuo et al. [55] reported a 2.2 times enhancement of flexural strength when 0.4 wt.% CNFs were added to MgO-C refractories. Zhu et al. [50,51] reported that MgO-C refractories containing homogeneous distributions of nano-carbons, especially CNTs and CB have a higher residual cold modulus of rupture (CMOR) and lower strength loss than MgO-C conventional refractories after thermal shock since nano-scaled materials can absorb and relieve the stress due to the thermal expansion and shrinkage of refractory particles. Moreover, CNTs and CB contribute to reducing the misdistribution of thermal stress in the MgO-C refractories.

As can be noted, due to their excellent physical, chemical, and mechanical properties, carbon nanotubes have attracted the attention and interest of researchers since their discovery in 1991. The CNTs (single or multi-walled) have been referred to as the material of the 21st century, due to their unique characteristics such as high elastic modulus (1 TPa) and tensile strength (150 GPa) compared to the existing fibers [66,67]. However, adequate dispersion of carbon nanotubes in the refractory still represents an important challenge due to their high specific surface areas. In addition to this, the relatively high cost of nanotubes, which depends on certain characteristics, such as parameters of synthesis and composition of the catalyst, represents an obstacle to promoting their applications on an industrial scale. The greater the purity and finer diameter of the carbon nanotubes, the greater the cost [68].

Following the approach of using nanomaterials in the design and development of refractory materials, researchers consider the possibility of improving physical, mechanical, and chemical properties at high temperatures if nanoparticles are added properly [69,70]. In refractory castables, nanoparticles fill gaps and generate fast diffusion paths to remove water particles. This leads to an increase in surface energy and improves the particle packing of the system. This certainly has a positive effect on properties such as oxidation, corrosion, and thermal shock resistance. In addition, recent studies have shown that nanopowders and colloidal suspensions have improved the bond-nature in refractory castables, which leads to the use of lower sintering temperatures to achieve better densification [71].

In the steel industry, there is also a tendency to use refractory castables with high alumina and spinel contents. Both Al_2_O_3_-spinel and Al_2_O_3_-MgO refractories are widely used as a steel ladle lining below the slag line, although in recent years the Al_2_O_3_-MgO refractories have been replaced by Al_2_O_3_-spinel due to their superior properties and their lower cost [69]. Several studies conducted in recent years have been aimed at using nanomaterials in the compositions of spinel-containing refractories because spinel-containing refractories possess superior thermal shock resistance, a high melting point, and high chemical stability, which undoubtedly allows them to be an option in many of the industrial applications [71,72].

Additionally, researchers have studied refractory matrices of MgO with additions of nanoparticles improving their properties, for example, Ghasemi-Kahrizsangi et al. studied the impact of adding Al_2_O_3_ [23] and ZrSiO_4_ [56] nanoparticles on the properties of MgO-C refractories. Nano-Al_2_O_3_ addition promoted the densification of MgO-C refractory due to the formation of MgAl_2_O_4_, AlN, and Al_4_C_3_ phases; nano-Al_2_O_3_ also improved the oxidation resistance of the MgO-C refractories. Furthermore, they found out that nano-ZrSiO_4_ improved the hydration resistance, the optimum content was 2 wt.%, obtaining a maximum flexural strength of 244 kg/cm^2^. Zagar et al. [57] studied the effect of the particle size of Cr_2_O_3_ on the densification of magnesia refractories. The results showed that as the particle size of Cr_2_O_3_ was reduced (≈20 nm), the density of the MgO refractories was enhanced at relatively low temperatures (≈850 °C). Azhari et al. [58] investigated the effect of the addition of nano-Fe_2_O_3_ on the magnesia-chrome refractory matrix; they found that the dissolution of nano-Fe_2_O_3_ and ionic migration improved the sintering process as well as the direct bonding structure. Huizhong et al. [59] also studied the addition of nano-Fe_2_O_3_ on the magnesia-chrome refractory matrix. They reported that the sintering temperature can be reduced (≈150 °C) [10]. Chen et al. [60] studied MgO-CaO refractories with the addition of ZrO_2_ micro- and nano-powders; their results showed that the densification was promoted by the addition of nano-ZrO_2_, which led to the formation of CaZrO_3_, thereby enhancing the thermal shock resistance and the slag corrosion resistance. Das [35] studied the effect of micro- and nano-spinel on MgO-C refractories sintered at 1000 °C. The results showed that adding 1 wt.% nano-spinel obtained superior properties compared to the sample containing 10 wt.% of micro-spinel. The effect of MgAl_2_O_4_ and Cr_2_O_3_ nanoparticles addition on the properties of MgO-CaO refractories was studied by Salman Ghasemi-Kahrizsangi et al. [61,62]. Cr_2_O_3_ nanoparticles improved the hydration resistance due to the formation of high hydration resistance phases such as CaCr_2_O_4_ and MgCr_2_O_4_. Adding spinel nanoparticles led to the appreciable improvement of the slaking resistance of the refractories as well as the achievement of a higher matrix densification.

Taking the above into account, the present research work is a complement to previous research work [65], whose aim is to investigate the effect of α-Al_2_O_3_ nanoparticles content on the thermo-mechanical properties and microstructural evolution of an ultra-low carbon MgO refractory sintered at 1300, 1500, and 1600 °C.

## 2. Experimental Procedure 

**Figure 1:**
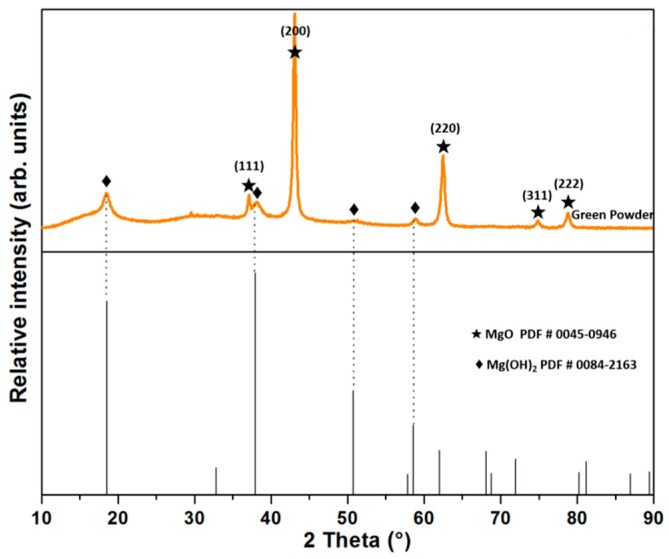
X-ray diffraction (XRD) patterns of MgO as raw material (green powder).

Industrial-grade magnesia (MgO) with high purity (provided by Magnelec Industries) and high-grade nano-alumina oxide (α-Al_2_O_3_) in α polymorphic phase were used as raw materials in this investigation. The chemical composition of the MgO (with a mean particle size <45 µm) was determined by a Philips Analytical X-ray fluorescence (XRF) spectrometer, (model epsilon 1, Malvern Panalytical, Westborough, MA, USA), and it is specified in Table 1. Figure 1 displays the X-ray diffraction (XRD) patterns of green powders of MgO where the main peaks correspond to MgO, while the weak peaks correspond to Mg(OH)_2_ or brucite phase (PDF #84-2163). The formation of Mg(OH)_2_ is attributed to the reaction of active MgO with the moisture environment, which is frequently observed in industrial-grade raw materials.

Table 2 provides the main characteristics of α-Al_2_O_3_ nanoparticles with a mean particle size of 50 nm supplied by Skyspring Nanomaterials (USA). The size, structure, and morphology of α-Al_2_O_3_ nanoparticles were examined by transmission electron microscopy (TEM, model G2 80-300, FEI Company, Hillsboro, OR, USA).

As it is well known, one of the most critical issues related to the use of nanoparticles is their dispersion. The nanoparticles were homogeneously distributed in the MgO matrix, as follows: a dispersed suspension of α-Al_2_O_3_ nanoparticles (X wt.% α-Al_2_O_3_ nanoparticles, X = 1, 3, or 5) was elaborated using a copolymer dispersant (10 wt.% Zephrym PD 3315 in relation to the wt.% of nanoparticles) and 3 wt.% of acetone in relation to the wt.% of MgO as a wet medium. Magnetic stirring was used for 10 min, then the solution was placed for 1 h in an ultrasonic dispersion equipment (Aquasonic TM 75T) at maximum speed. Afterward, the solution was poured into the MgO powders and homogenized using a mechanical mixer (Alghamix II-Zhermack) for 15 min at 100 rpm. Then, the mixture was placed in a steel die and uniaxially pressed under 100 MPa for 2 min using a Dogo Tuls press, to shape cylindrical samples (25.4 mm diameter and about 25.4 mm height).

The refractory mixtures were made according to the batch compositions given in Table 3. In the same table, the green densities are shown to compare them with densities after the sintering process. 

The green samples were dried at 120 °C for 24 h. After the drying process, the specimens were sintered in a Lindberg/Blue M (BF51524C model) electric furnace at 1300, 1500, and 1600 °C with a heating rate of 5 °C/min and soaked for 4 h at the designated temperature. Sintering parameters were based on literature [15,23,42,44,45,56,61,65]. The phase composition was analyzed using an X-ray diffractometer (XRD; Bruker D8 Advance model) with Cu Kα radiation (λ = 1.5406 Å) operated at 40 kV and 30 mA. The scans were performed in the 2θ range from 10 to 90° with a scan step of 0.05° and 1.5 s per step in continuous mode. The bulk density (BD) and apparent porosity (AP) of sintered samples were evaluated by the Archimedes method (ASTM-C20). The mechanical resistance was determined by the cold crushing strength method (CCS). A mechanical testing machine (ELE-International, ABR-AUTO model) was used. In addition, the micro-hardness was evaluated by the Vickers technique (HV) using a Shimadzu microhardness tester. The loading time was 10 s with a loading force set as 2.94 N. Specimens 25.4 mm in diameter and 25.4 mm in height were used in both evaluations and the reported values are the average of 15 measurements for each designed composition. The microstructure of the refractory samples was studied using a FEI Nova NanoSEM 200 scanning electron microscope (SEM) equipped with an electron dispersive X-ray (EDX) detector (EDAX, Apollo XP 2930 model).

The refractory compositions (A0, A1, A3, and A5) were subjected to simultaneous differential scanning calorimetry (DSC)/thermogravimetric (TGA) analyses using a simultaneous TGA-DSC model Q600 instrument to evaluate the thermal events related to the α-Al_2_O_3_ nanoparticles addition during the sintering process up to 1000 °C.

## 3. Results and Discussion

**Figure 2:**
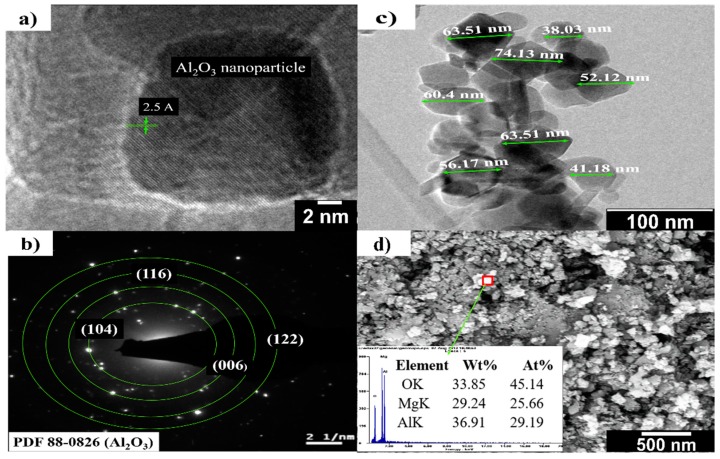
(**a**) TEM image of an α-Al_2_O_3_ nanoparticle; (**b**) electron diffraction pattern of an α-Al_2_O_3_ nanoparticle; (**c**) TEM image of α-Al_2_O_3_ nanoparticles sizes; (**d**) SEM image of α-Al_2_O_3_ nanoparticles in MgO matrix.

Figure 2a shows a TEM image corresponding to the α-Al_2_O_3_ nanoparticles used in this investigation. Figure 2b shows the selected area electron diffraction (SAED) patterns of α-Al_2_O_3_ nanoparticles. This pattern matches the standard pattern of α-alumina (α-Al_2_O_3_) (PDF#88-0826). Figure 2c shows the sizes of α-nano-Al_2_O_3_, with quasi-spherical particles with an average size of 55 nm, which can be observed one above the other. The SEM-EDX data in Figure 2d confirms a suitable dispersion of α-Al_2_O_3_ nanoparticles into the MgO matrix in the A5_16_ green sample, i.e., before the sintering process.

**Figure 3:**
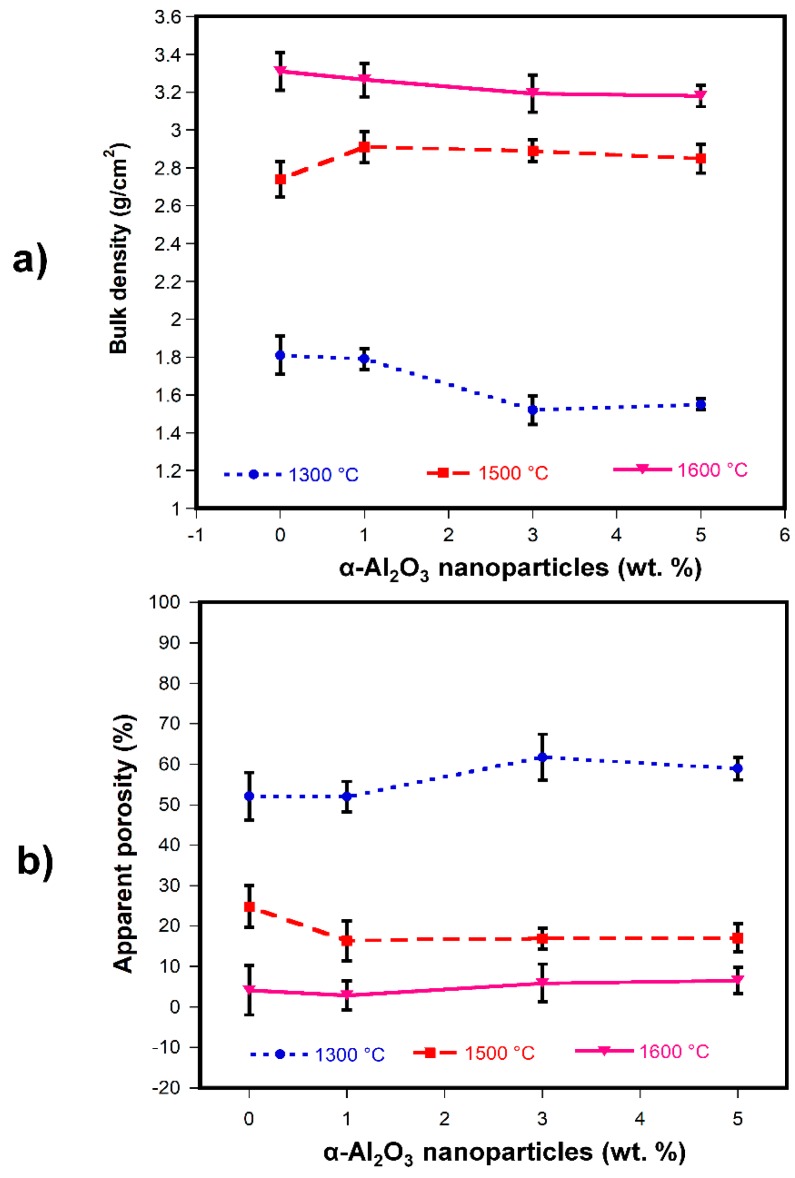
(**a**) Variation of the bulk density and (**b**) variation of the apparent porosity of refractory magnesia as a function of the content of α-Al_2_O_3_ nanoparticles.

Figure 3a,b shows the results of bulk density and apparent porosity of sintered samples at 1300, 1500, and 1600 °C (according to the relation of samples shown in Table 3). In Figure 3a, it can be seen that the bulk density of the refractory compositions gradually increases compared to the green densities with the increase in sintering temperature, specifically at 1500 and 1600 °C. 

On the other hand, as can be seen from Figure 3a, there is a decrease in bulk density of about 10% for the refractory samples sintered at 1300 °C compared to the green density values.

This phenomenon is related to some thermal events of mass loss of the brucite phase (detected by XRD analysis), which is explained as follows: first, about 10% mass loss related to adsorbed moisture occurs between 50 and 115 °C. Then, a mass loss of about 23% due to dihydroxylation occurs between 315 and 450 °C. Finally, a mass loss of about 3.5% due to the diffusion of water steam occurs between 450 and 1000 °C. Therefore, a mass loss of about 30% is related to the moisture loss from the brucite phase, which leads to a highly porous and microcracked microstructure, as reported in the literature [73]. In addition, perhaps the temperature of 1300 °C is too low to induce optimum densification since between 800 and 950 °C, the brucite hexagonal structure tends to convert into a cubic magnesia one, followed by the beginning of the sintering process above 1200 °C [74].

It is well known that bulk density depends strongly on the temperature; as the temperature increases, the diffusion of species takes place, which increases the neck between particles and eliminates the porosity resulting in a denser ceramic body. However, in samples sintered at 1300 and 1600 °C, a tendency where the bulk density decreases as the content of α-Al_2_O_3_ nanoparticles is increased in the refractory compositions was observed.

This phenomenon can be attributed to the formation in-situ of MgAl_2_O_4_ spinel since it was extensively reported in literature that the lower expansion of spinel versus magnesia (MgO) leads to the formation of microcracks that affect the bulk density, besides this phenomenon can be more detrimental if the grain size of spinel is sufficiently large to form larger cracks that lead to high porosity [23]. This effect is more pronounced at 1300 °C since at this temperature adequate sintering of the refractory body has not been achieved, together with the micro-cracking caused by the in-situ formation of the MgAl_2_O_4_ spinel [72]. Furthermore, microcrack formation and larger spinel grains with larger cracks can be combined at 1600 °C resulting in the decrement in bulk density. It is important to mention that when the matrix has a good dispersion and a controlled size of these microcracks, they can act as crack arrestors improving the mechanical resistance of the refractory bodies. At 1500 °C, the bulk density increases as the α-Al_2_O_3_ nanoparticles content increases considering the reported value reached by the A0_15_ composition (2.74 g/cm^3^). As was discussed above, with a controlled spinel grain growth better matrix densification can be achieved. The maximum value of bulk density was 3.31 g/cm^3^, which corresponded to the A0_16_ sample sintered at 1600 °C.

In Figure 3b, a decrease in the apparent porosity can be observed as the sintering temperature is increased in all the refractory compositions; however, the apparent porosity increased as the α-Al_2_O_3_ nanoparticles content increased. This behavior is accomplished by samples sintered at 1300 and 1600 °C. This phenomenon is explained by the observation that in situ spinel formation in the MgO matrix (usually achieved at temperatures within 1000 to 1200 °C) causes microcracks to form and results in an increase of apparent porosity. Similar behavior in apparent porosity was reported by Ghasemi-Kahrizsangi et al. [61]. In their research work, MgAl_2_O_4_ nanoparticles were added in different percentages (0–8 wt.%) to MgO-CaO refractories and sintered at 1650 °C. The lowest apparent porosity value was achieved by adding 6 wt.% of MgAl_2_O_4_ nanoparticles, followed by an increase in apparent porosity with 8 wt.% MgAl_2_O_4_ nanoparticles. With a higher content of MgAl_2_O_4_ nanoparticles and due to the large difference in thermal expansion coefficients between MgO and MgAl_2_O_4_, excessive micro-cracking was generated, which caused the increase in apparent porosity.

At the sintering temperature of 1500 °C, the apparent porosity decreased as the content of α-Al_2_O_3_ nanoparticles increased and remained almost constant at 1, 3, and 5 wt.% of α-Al_2_O_3_ nanoparticles addition. It is possible that at 1500 °C, the spinel appears in the triple points leading to better densification, i.e., a lower porosity is reached as was observed in Figure 3b.

The minimum value of apparent porosity was 3% corresponding to the A1_16_ sample sintered at 1600 °C. In this case, perhaps a well-dispersed spinel and mostly located in the triple points can lead to this behavior.

**Figure 4:**
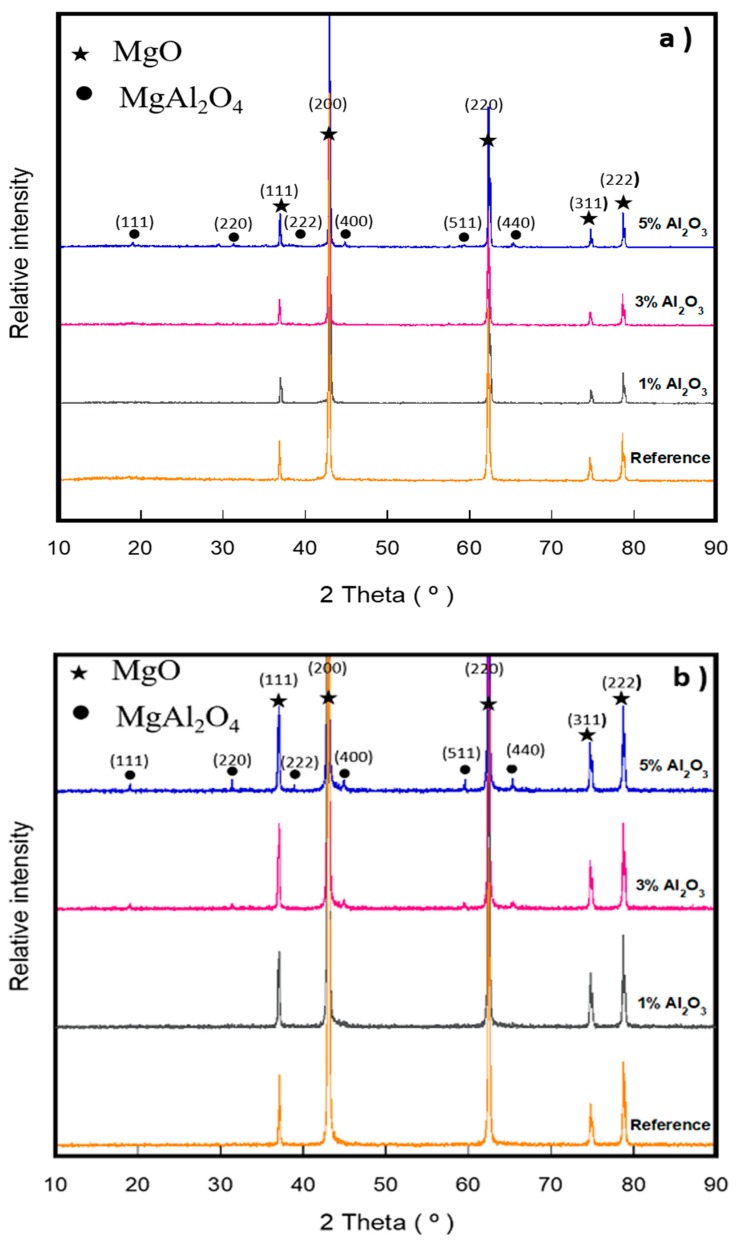

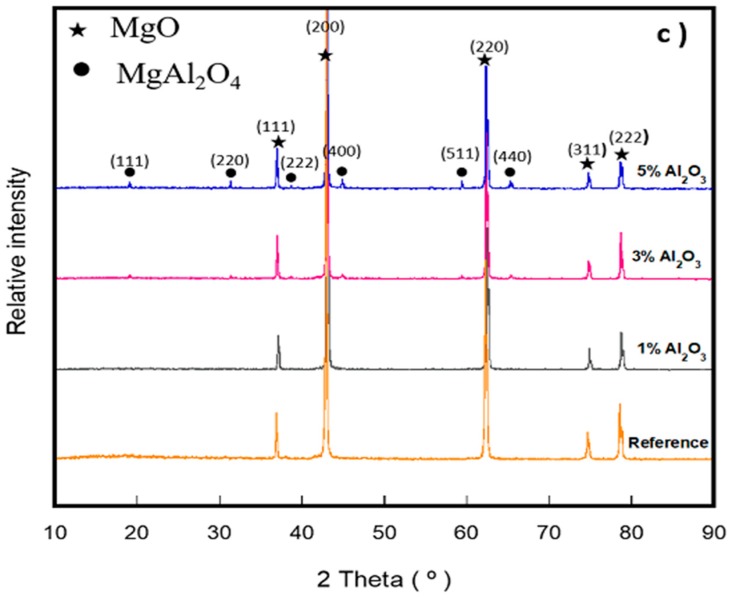
XRD diagrams of the samples containing different levels of α-Al_2_O_3_ nanoparticles at different temperatures: (**a**) 1300 °C, (**b**) 1500 °C, and (**c**) 1600 °C.

Figure 4a–c shows the XRD results of samples sintered at 1300, 1500, and 1600 °C (according to samples shown in Table 3). For reference, pure magnesia was also plotted. For all the refractory compositions (including the reference composition) at all sintering temperatures, reflections from the (111), (200), (220), (311), and (222) planes that match the standard pattern for MgO (PDF#0045-0946) can be seen. Tricalcium silicate (C_3_S) and dicalcium silicate (C_2_S) do not occur since high-grade purity magnesia was used; these are bonding phases that can usually be detected.

For all refractory compositions with the addition of α-Al_2_O_3_ nanoparticles, reflections that correspond to the MgO phase (PDF# 0045-0946) can be seen. On the other hand, depending on the specific α-Al_2_O_3_ nanoparticles addition and sintering temperature, it was possible to detect the reflections with different intensity from the (111), (220), (222), (400), (511) and (440) planes that match the standard pattern of MgAl_2_O_4_ (PDF#0086-2258). As expected, the amount of spinel phase increased with higher temperatures.

For A5_13_, A5_15_, and A5_16_ samples, strong reflections from the (111), (200), (220), (311), and (222) planes that correspond to the MgO phase (PDF#0045-0946) can be seen. Additionally, there are weak but easily detectable reflections at (111), (220), (222), (400), (511), and (440) planes that match the standard pattern of MgAl_2_O_4_ (PDF#0086-2258). Comparing the intensities of the peaks for MgO and MgAl_2_O_4_, it can be seen that the major phase corresponds to MgO, and the MgAl_2_O_4_ is present as a second phase. These two phases were corroborated by SEM and EDX analysis

Table 4 shows the results of the quantitative phase estimation of crystalline phases for all the experimental compositions that were carried out by the relative intensity method. It was found that the A5_15_ composition contains the highest concentration of spinel, which is 9.97 wt.%.

**Figure 5:**
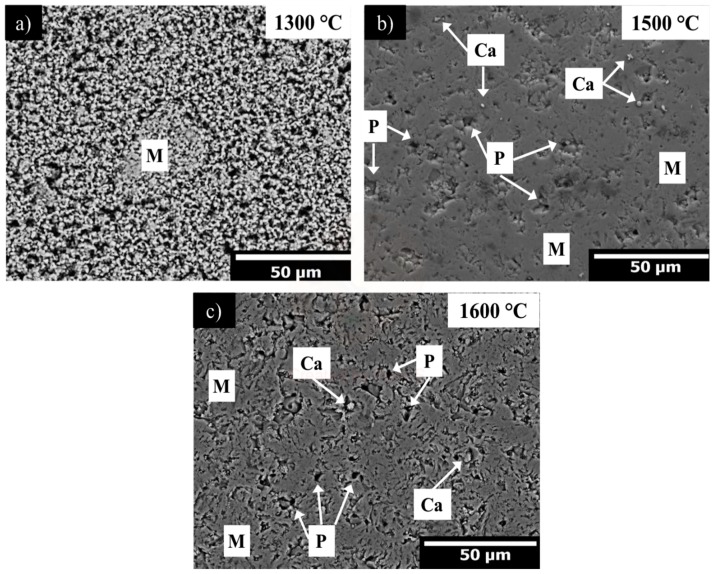
SEM micrograph in back-scattered electron image mode corresponding to a typical microstructure of magnesia, sintered at (**a**) 1300 °C, (**b**) 1500 °C, and (**c**) 1600 °C. Where, M = magnesia, Ca = CaO, P = porosity.

Figure 5 shows the microstructure corresponding to the A0_13_, A0_15_, and A0_16_ sintered samples at (a) 1300, (b) 1500, and (c) 1600 °C respectively.

Figure 5a shows a microstructure where the contact points between adjacent MgO particles with necking formation are recurrent, besides high porosity are detected (≈50%). These microstructural characteristics originate due to the use of low sintering temperature, that it is not sufficient to obtain a dense MgO microstructure. For the A0_15_ sample, a denser microstructure than the A0_13_ sample (2.74 g/cm^3^ compared to 1.8 g/cm^3^, respectively) with some free lime particles was observed as it is indicated in Figure 5b. Figure 5c shows the densest microstructure corresponding to the A0_16_ sample (3.31 g/cm^3^), with closed porosity. By EDX analysis, CaO (impurity from raw material) and MgO phases were detected. In addition, by an image analyzer software (Gatan Microscopy Suite-GMS), the mean grain size of the MgO in A0_13_, A0_15_, and A0_16_ sintered samples corresponding to 3, 5, and 10 µm, respectively, was determined.

Low melting point phases as monticellite (CaMgSiO_4_) and merwinite (Ca_3_MgSi_2_O_8_) with initial liquid formation at 1490 and 1575 °C, respectively, not only could be helpful in the material densification process but also could have a negative effect since at high temperature these phases can lead to the material softening. Therefore, the CaO/SiO_2_ ratio in an MgO matrix is extremely important. In this research, the CaO/SiO_2_ ratio is 1.5, which prevents the formation of low melting point phases [65].

**Figure 6:**
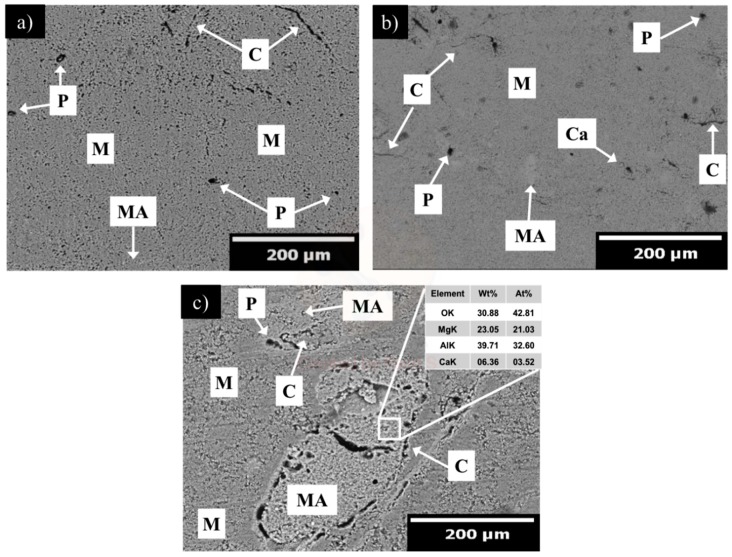
SEM micrograph of microstructural evolution of magnesia samples with increasing addition of α-Al_2_O_3_ nanoparticles, sintered at 1300 °C. (**a**) 1, (**b**) 3, and (**c**) 5 wt.%. M = magnesia, C = microcracks, P = pores, and MA = MgAl_2_O_4_ spinel.

**Figure 7:**
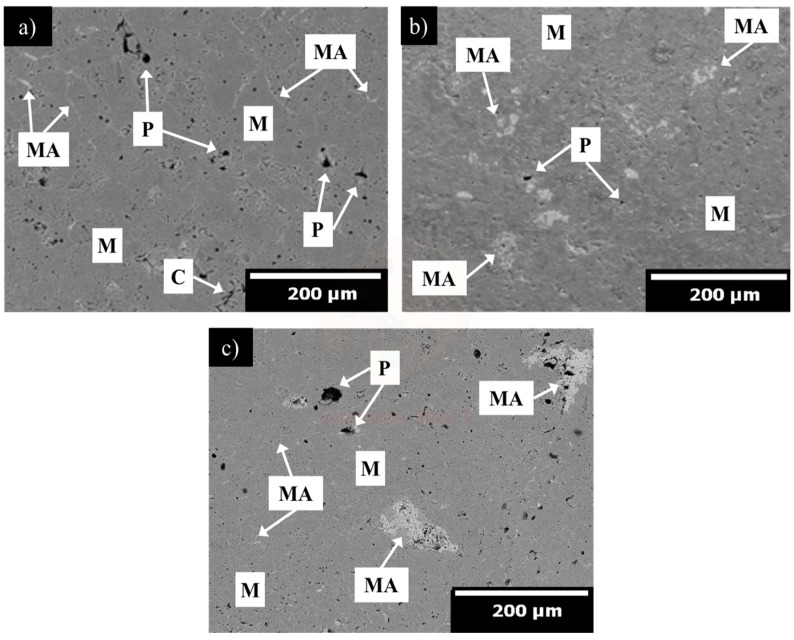
SEM micrograph of microstructural evolution of magnesia samples with increasing addition of α-Al_2_O_3_ nanoparticles, sintered at 1500 °C. (**a**) 1, (**b**) 3, and (**c**) 5 wt.%. M = magnesia, C = microcracks, P = pores, and MA = MgAl_2_O_4_ spinel.

**Figure 8:**
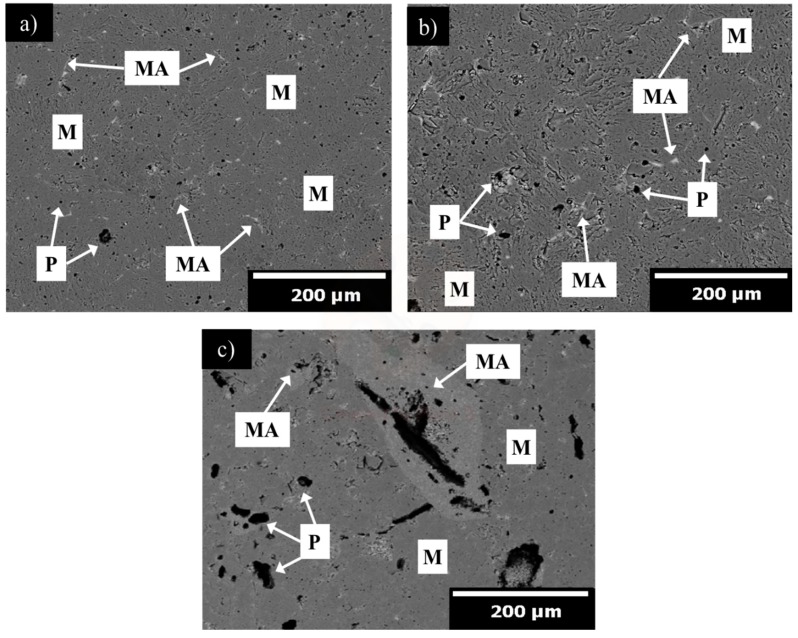
SEM micrograph of microstructural evolution of magnesia samples with increasing addition of α-Al_2_O_3_ nanoparticles, sintered at 1600 °C. (**a**) 1, (**b**) 3, and (**c**) 5 wt.%. M = magnesia, P = pores, and MA = MgAl_2_O_4_ spinel.

Figure 6, Figure 7 and Figure 8 show the microstructural evolution of magnesia samples with increasing addition of α-Al_2_O_3_ nanoparticles (1, 3, and 5 wt.%) sintered at 1300 (Figure 6a–c), 1500 (Figure 7a–c), and 1600 °C (Figure 8a–c).

Figure 6a shows the microstructure corresponding to the sample with 1 wt.% addition of α-Al_2_O_3_ nanoparticles. A highly porous microstructure (≈52%), where quasi-spheroidal, small (<3 µm), and homogeneously distributed pores were observed. The dark grey phase corresponds to magnesia. Few spinel particles were detected by the EDX analysis with a mean grain size of around 11 µm. As the addition of the α-Al_2_O_3_ nanoparticles is increased, microcracks are generated, as shown in Figure 6b,c. In Figure 6b a high porosity in the microstructure is recurrent (≈62%). In the A3_13_ microstructure, cracks are more frequent comparing to those observed in the A1_13_ microstructure. This microstructural characteristic can be the reason for an increment in the porosity percentage. The spinel population incremented as the addition of α-Al_2_O_3_ nanoparticles was increased. Spinel particles with a mean grain size of around 25 µm were detected. In Figure 6c a porous microstructure is also observed (≈60%). Spinel particles with a mean particle size of around 125 µm were detected. Here, it can be observed that initial spinel formation occurred around the periphery of the alumina particles and proceeded towards the particle center. Diametric cracks around the spinel are observed. Some of them are above 200 µm in size; therefore, this issue can be catastrophic since it can lead to a weak refractory microstructure.

It is well known that MgAl_2_O_4_ spinel formation is accompanied by a 5%–7% volume expansion, which contributes to the microcrack generation. In addition, the large difference in thermal expansion coefficient between MgO (13.6 × 10^−6^ °C^−1^ from 25 to 1000 °C) and MgAl_2_O_4_ spinel (8.4 × 10^−6^ °C^−1^ from 25 to 1000 °C) generates very large hoop tensile stresses around spinel particles, which produce extensive microcracking. These microcrack networks developed around spinel particles may also either be barriers to subsequent crack propagation in service or allow stress relief during heating. Thus, crack propagation is a much greater energy consumption process than crack initiation in the magnesia-spinel matrix. 

In Figure 7a–c, the MgO phase with well-defined grain boundaries (dark grey particles) can be observed. Additionally, a reduction in porosity can be seen compared with the refractory samples sintered at 1300 °C; this means that at higher sintering temperatures the diffusion rate increases, which lowered porosity and created an effective densification process. 

In Figure 7a, a microstructure with around 16% of porosity and quasi-spherical pores (d_50_ = 5 µm) are observed. Spinel particles (light grey particles) with a mean grain size of around 12 µm were found through the grain boundary and triple points. In Figure 7b, the microstructure corresponding to the A3_15_ sample composed of a magnesia matrix (dark grey particles) and spinel grains (light grey particles) well-distributed in the magnesia matrix is observed. In addition, quasi-spherical pores can be observed homogenously distributed in the MgO matrix, with a mean size of 5 µm. Spinel particles with a mean particle size of around 25 µm were detected through the entire matrix. In Figure 7c, the microstructure corresponding to the A5_15_ sample with a similar porosity registered in the A3_15_ sample (≈17%) can be observed. Once again, quasi-spherical pores are observed in the size range of 5 to 20 µm. The MgAl_2_O_4_ spinel is clearly observed as the light grey particles. Most of these particles are in an agglomerated state with a size range of 40 to 80 µm. Despite these agglomerations, no large cracks were founded around the spinel particles. This microstructural characteristic is beneficial since spinel can be acting as a matrix reinforcement phase.

In Figure 8a,b, a dense magnesia matrix can be seen. Pores are mostly quasi-spherical in shape with a mean size of <5 µm. In both figures, the spinel formation is observed near the grain boundary and triple points, since these specific places can act as nuclei sources. Spinel particles with a mean particle size around 12 and 20 µm for A1_16_ and A3_16_, respectively, were detected through the entire matrix. In Figure 8c, spinel agglomeration (above 100 µm in size) it is observed, besides a strongly bonded peripheral spinel and a hollow core can be seen, as indicated in Figure 8. This microstructural characteristic is claimed to give better fracture toughness [75]. However, large cracks (above 200 µm in size) as are presented in the A5_16_ microstructure are detrimental to the mechanical resistance. The matrix densification mechanism was evidently promoted at the temperature of 1500 and 1600 °C. The use of the α-Al_2_O_3_ nanoparticles powder played a significant role in precisely controlling in situ spinel formation and effectively generating the development of microcrack networks around spinel particles. The microstructural analysis shows a strong correlation with the physical properties previously studied.

**Figure 9:**
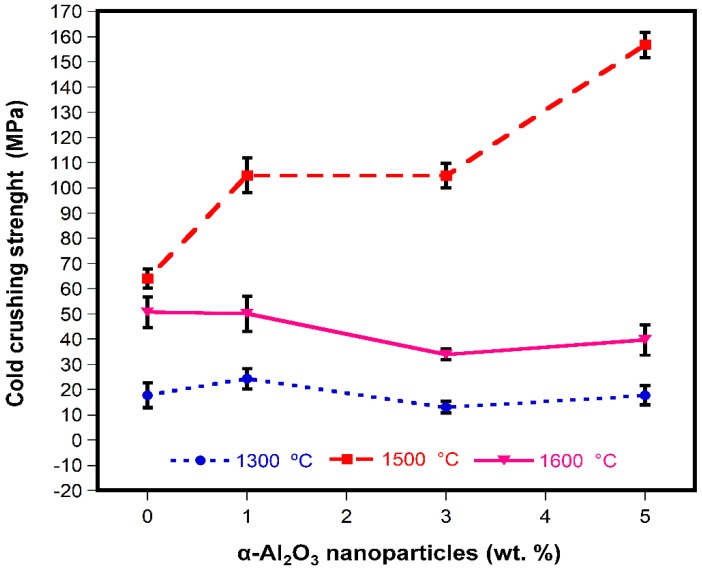
Cold crushing strength at different α-Al_2_O_3_ nanoparticles content.

Figure 9 shows the results of the cold crushing strength for different α-Al_2_O_3_ nanoparticle contents. It was found that at 1300 °C, the CCS remained almost unchanged with the increase in the content of α-Al_2_O_3_ nanoparticles, although at 1 wt.% of α-Al_2_O_3_ nanoparticles, an increase in mechanical resistance can be observed. This behavior can be explained in terms of matrix reinforcement by a second phase (spinel particles). However, the mechanical resistance of sintered refractory samples at 1300 °C is well below the reported values for both laboratory studies and commercial refractories, as shown in Table 5.

After sintering at 1500 °C, there was a significant improvement in mechanical resistance; the maximum value registered was 156 MPa and corresponded to the 5 wt.% of α-Al_2_O_3_ nanoparticles addition (A5_15_ sample). This reported mechanical resistance represents an enhancement of about 245%, considering the reference sample (A0_15_ = 64 MPa), and 79% considering the CCS values reported for commercial refractories.

The mechanical resistance achieved by the A5_15_ sample represents a double benefit since excellent mechanical properties are obtained and the lowest sintering temperature is used, taking into account that the sintering temperatures of MgO-based refractories range between 1500 and 1800 °C, which represents an important technological advance [1,2].

On the other hand, at the sintering temperature of 1600 °C, when the α-Al_2_O_3_ nanoparticles content increased from 1 to 5 wt.%, the CCS decreased, although the mechanical resistance obtained for samples A0_16_ and A1_16_ is within that reported for commercial MgO-based refractories. 

This behavior can be attributed to the formation of MgAl_2_O_4_, which resulted in the formation of microcrack networks around this phase, due to the large difference in the thermal expansion coefficients between MgO and MgAl_2_O_4_. These microcracks are beneficial (up to a certain limit) for the mechanical properties, helping to dissipate the stored energy in compression load, as shown in the samples tested at 1500 °C [76]. However, when the sintering temperature increased to 1600 °C, the size and numbers of microcracks also increased, which had a detrimental effect by reducing the overall strength and stiffness of the refractory samples. As it is observed, the diminish resistance for the A0_16_ sample could seem strange since the maximum sintering temperature and no spinel addition was used but it is important to remember that the magnesia matrix becomes very stiff as the sintering temperature increases. Aksel et al. have reported that spinel grain growth takes place around 1100 °C, followed by a significant increase from 1500 to 1625 °C; subsequently, grain growth stops above 1700 °C [77]. The growth of grain in the MgO matrix leads to a decrease in mechanical resistance because there is no crack interruption, the above mentioned is reflected detrimentally in terms of mechanical resistance; therefore, a second phase must be added to permit a better microstructural flexibly and an improvement in mechanical resistance.

**Figure 10:**
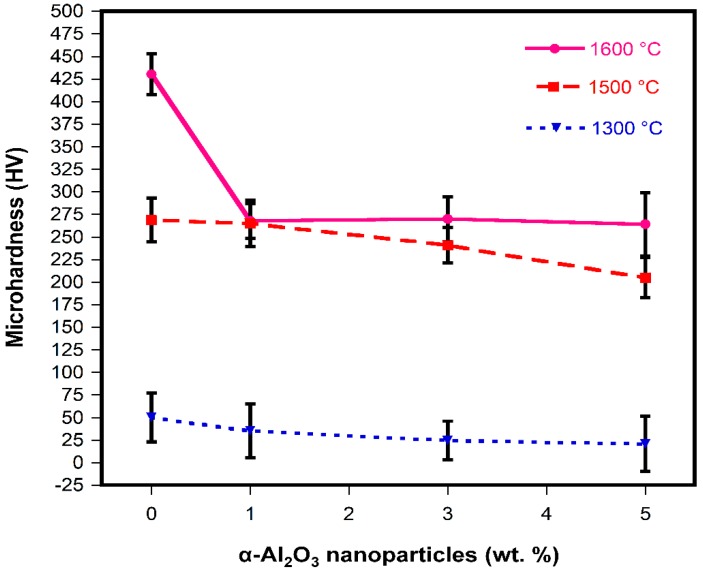
Variation of microhardness with α-Al_2_O_3_ nanoparticle additions.

On the other hand, hardness helps to characterize resistance to deformation, densification, and fracture [80]. Ceramics’ hardness depends on their chemical composition and the following microstructure characteristics: porosity, grain size, and grain-boundary phases. Figure 10 shows the relationship between microhardness and α-Al_2_O_3_ nanoparticles content at 1300, 1500, and 1600 °C, respectively. The analysis of the results clearly shows that an increment in sintering temperature led to an increase in hardness. These results are well correlated with the specimen microstructure; mostly due to the densification, since the denser the matrix is the harder specimen is. At 1300 °C, a reduction in microhardness can be observed as α-Al_2_O_3_ nanoparticles were added. This phenomenon can be related to the porosity that originated during spinel formation. At 1500 °C, the specimens reached HV values almost three times larger than specimens sintered at 1300 °C. According to this observation, the higher the sintering temperature, the higher the hardness values reached. However, at 1500 °C, a reduction in HV hardness can be observed as α-Al_2_O_3_ nanoparticles were added. This tendency is similar to that registered in the specimens sintered at 1300 °C. This phenomenon is also attributed to the spinel formation. The maximum hardness value was 430 HV corresponding to the A0_16_ specimen sintered at 1600 °C; at this sintering temperature, a sharp decrease in HV hardness was observed after α-Al_2_O_3_ nanoparticles were added (1 wt.%) followed by a negligible change in HV hardness as α-Al_2_O_3_ nanoparticles were increased. This behavior can be explained as follows: as the sintering temperature increases, the MgO matrix develops with an elevated hardness; at this moment is very important to remember that one of the disadvantages of magnesia is the high stiffness developed since at high stresses it becomes brittle. On the other hand, one of the aforementioned advantages when second phases are used as the spinel is the microstructural flexibly reached that permits a better mechanical property [78]. Therefore, at 1600°C as the α-Al_2_O_3_ nanoparticles were added a diminish in hardness was expected, while an improvement in microstructural flexibility was reached.

**Figure 11:**
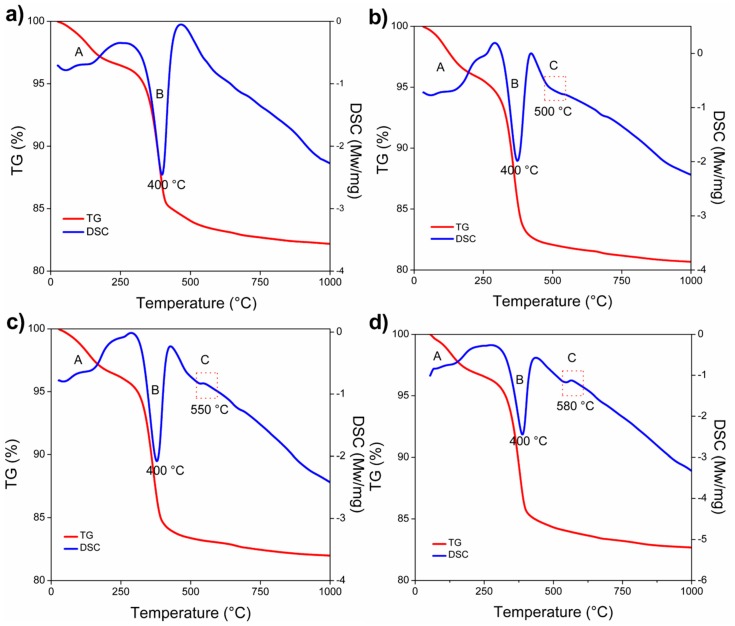
DSC-TGA results of samples: (**a**) MgO, (**b**) 1 wt.% Al_2_O_3_, (**c**) 3 wt.% Al_2_O_3_, and (**d**) 5 wt.% Al_2_O_3._

Figure 11a–d shows the DSC-TGA thermograms of A0, A1, A3, and A5 refractory samples, respectively. Several thermal events are identified in the analysis up to 1000 °C, which involved the evaporation of H_2_O, brucite decomposition, and spinel formation.

In all thermograms, a thermal event identified as peak A is presented at about 100 °C, which is related to the evaporation of H_2_O. Intense weight loss (≈17 wt.%) occurred in all tested specimens between 300 and 425 °C (a thermal event label as peak B), which is associated with the dehydration of the MgO; i.e., the brucite decomposition. MgO apparently absorbed some environmental moisture due to its hygroscopic nature. A small exothermic peak at 550 °C [81] that does not appear in the reference thermogram (A0) is assigned to the nucleation and formation of spinel by the reaction between alumina and magnesia (peak C). According to the literature, some authors claimed a spinel formation at a temperature lower (about 550 °C) than the one presented in this investigation [64,81,82,83,84,85]. Although, thermodynamically the formation of spinel becomes possible at 550 °C, there are many factors as temperature, particle size, concentration, and time, among other factors to reach a whole formation of in situ spinels [64,75,81,82,83,84,85].

## 4. Conclusions

- The sintering temperatures and the α-Al_2_O_3_ nanoparticle content had an important role in the physical and mechanical properties of MgO-based refractories.
- DSC-TGA analysis allowed to relate a small exothermic peak at 550 °C, with the nucleation and formation of the spinel (MgAl_2_O_4_) due to the reaction between α-Al_2_O_3_ nanoparticles and magnesia.
- The adequate dispersion and presence in triple points of the spinel phase (MgAl_2_O_4_) promoted the densification of the magnesia matrix significantly at sintering temperatures of 1500 and 1600 °C.
- The use of α-Al_2_O_3_ nanoparticles played significant roles in precise control in situ spinel formation and to effectively develop microcrack networks around spinel particles.
- The large difference in thermal expansion coefficient between MgO and MgAl_2_O_4_ led to the formation of microcracks, which can be beneficial up to a certain limit because they allow dissipating the stored energy in compression loads.
- The maximum CCS value registered was 156 MPa, which corresponded to the addition of 5 wt.% of α-Al_2_O_3_ nanoparticles at 1500 °C.
- The size and number of microcracks were increased at the sintering temperature of 1600 °C, which had a detrimental effect since the overall strength and stiffness of the refractory samples were reduced.
- The physical and mechanical properties developed by the refractories studied in this research work are comparable and/or superior to those of MgO-based commercial refractories, which allows them to be considered as an option for application in the steelmaking industry.

## Figures and Tables

**Table 1:** Chemical composition (wt.%) of magnesia.

MgO	CaO	Fe_2_O_3_	SiO_2_	LOI
97.43	0.9	0.06	0.6	1.01

**Table 2:** Characteristics of the high-grade of α-Al_2_O_3_ nanoparticles (SSA, specific surface area).

Purity (wt.%)	Size (nm)	SSA (m^2^/g)	Color
99.9	50	18	White

**Table 3:** Sintering temperature, sample codes, batch compositions with its green densities and samples used in differential scanning calorimetry (DSC) analysis.

Temperature	Sample Code	Batch Composition (wt.%)		Green Density (g/cm^3^)
		MgO	Al_2_O_3_	
1300 °C	A0_13_	100	0	2.29
	A1_13_	99	1	2.32
	A3_13_	97	3	2.29
	A5_13_	95	5	2.32
1500 °C	A0_15_	100	0	2.32
	A1_15_	99	1	2.38
	A3_15_	97	3	2.32
	A5_15_	95	5	2.32
1600 °C	A0_16_	100	0	2.31
	A1_16_	99	1	2.29
	A3_16_	97	3	2.32
	A5_16_	95	5	2.31
1000 °C (DSC)	A0	100	0	2.36
	A1	99	1	2.32
	A3	97	3	2.34
	A5	95	5	2.31

**Table 4:** Quantitative phase estimation of crystalline phases by the relative intensity method.

Phase	wt.%											
	Experimental Compositions											
	A0	A1_13_	A3_13_	A5_13_	A0_15_	A1_15_	A3_15_	A5_15_	A0_16_	A1_16_	A3_16_	A5_16_
**MgO**	100	99.69	99.02	96.07	100	97.22	94.19	90.03	100	98.87	96.28	90.37
**MgAl_2_O_4_**	0	0.310	0.980	3.93	0	2.78	5.81	9.97	0	1.13	3.72	9.63

**Table 5:** Physical and mechanical properties of MgO-based refractories from laboratory studies and commercial refractories.

Refractory Matrix	Modifying Agent	Density (g/cm^3^)	Apparent Porosity (%)	Maximum CCS (MPa)	Reference/Year
**Laboratory Study**					
MgO	nano-TiO_2_	3.46	0.52	234.7	[63]/2016
MgO	nano-Fe_2_O_3_/Al_2_O_3_	3.38/3.18	1.87/6.5	65.1/42.7	[65]/2015
MgO-C	nano-TiO_2_	3.32	4.97	42.6	[15]/2017
MgO-C	expanded graphite	3.05	3.0	59.7	[17]/2014
MgO-C	nano-Al_2_O_3_	3.31	5.73	40.2	[23]/2016
MgO-C	nano carbon black	3.12	4.25	51.0	[18]/2012
MgO-C	Fe nanosheets	-	-	92.4	[54]/2015
MgO-CaO	nano-Al_2_O_3_	3.5	5	100	[42]/2017
MgO-CaO	nano-SiO_2_	3.35	5.7	58.8	[44]/2017
MgO-CaO	nano-ZrO_2_/SiO_2_/TiO_2_	3.27/3.17/3.2	7.2/6.4/7.1	49/54/51	[45]/2018
MgO-CaO	nano-ZrSiO_4_	3.33	4.84	38.5	[56]/2017
MgO-CaO	nano-MgAl_2_O_4_	3.31	9.6	66.0	[61]/2017
MgO-CaO	nano-Cr_2_O_3_	3.35	2.9	82.3	[62]/2017
MgO-CaZrO_3_	Fe_2_O_4_	3.2	11.4	180.3	[78]/2015
**Commercial Refractories**					
MgO-iron spinel	FeAl_2_O_4_	2.9	16–17	45–55	[79]/2020
Magnesium aluminum-spinel	MgAl_2_O_4_	2.95	18–19	40–45	[79]/2020
Magnesia	CaO, SiO_2_	2.9	16–18	60	[79]/2020
Magnesia-zirconia	ZrO_2_	2.95–3.05	18–19	40–50	[79]/2020
Magnesia-chrome	Cr_2_O_3_	3.0–3.08	14–18	50–55	[79]/2020
